# Fetal Ovarian Cysts: Prenatal Diagnosis Using Ultrasound and MRI, Management and Postnatal Outcome—Our Centers Experience

**DOI:** 10.3390/diagnostics12010089

**Published:** 2021-12-31

**Authors:** Ioana Cristina Rotar, Stefania Tudorache, Adelina Staicu, Roxana Popa-Stanila, Roxana Constantin, Mihai Surcel, Gabriela Corina Zaharie, Daniel Mureşan

**Affiliations:** 1 1st Department of Obstetrics and Gynecology, “Iuliu Haţieganu” University of Medicine and Pharmacy Cluj-Napoca, 400012 Cluj-Napoca, Romania; cristina.rotar@umfcluj.ro (I.C.R.); mihai_surcel@yahoo.com (M.S.); muresandaniel01@yahoo.com (D.M.); 2 1st Clinics of Obstetrics and Gynecology Cluj-Napoca, Emergency County Clinical Hospital Cluj-Napoca, 400006 Cluj-Napoca, Romania; dr.roxana.constantin@gmail.com; 3 Department of Obstetrics Gynecology, Emergency County Hospital Craiova, 200642 Craiova, Romania; 4 Centre of Advanced Research Studies, Emergency County Hospital “IMOGEN”, 400006 Cluj-Napoca, Romania; roxanatania@yahoo.com; 5 Department of Radiology, “Iuliu Haţieganu” University of Medicine and Pharmacy Cluj-Napoca, 400012 Cluj-Napoca, Romania; 6 Neonatology Department, “Iuliu Haţieganu” University of Medicine and Pharmacy Cluj-Napoca, 400012 Cluj-Napoca, Romania; gabrielazaharie1966@gmail.com

**Keywords:** fetal ovarian cyst, fetal, prenatal ultrasound, MRI, ovarian torsion

## Abstract

The present study provides our clinical experience regarding the imaging diagnosis, management and postnatal outcome of neonates prenatally suspected of having developed ovarian cysts. This multicenter observational study included patients diagnosed prenatally with fetal ovarian cysts and follow-up in the postnatal period. Descriptive statistics were used to render the information regarding the prenatal imaging aspect of the fetal pelvic masses using ultrasound and/or MRI, prenatal surveillance and postnatal neonate’s immediate outcome, indications leading to surgery and pathologic aspect. The inclusion criteria were fulfilled by 21 patients. The mean gestational age at the time of initial diagnosis was 31.28 weeks of gestation (WG). Only five out of 21 cysts regressed completely during pregnancy without postnatal complications. In addition, 11 out of 21 infant’s required surgical treatment in the first two weeks after birth, mainly for ovarian torsion. Five out of 21 neonates were referred to postnatal follow-up clinically and by ultrasound, but three out of five cases required emergency surgical treatment for acute complications. Ultrasound plays a major role in the diagnostic of fetal ovarian cyst. From our experience, MRI does not bring supplementary data or change the management. Spontaneous resolution of fetal ovarian cysts is to be expected but the ovarian mass could lead to serious complications, if resolution does not occur in due time.

## 1. Introduction

Ovarian cysts are the most frequent type of abdominal tumor found in female fetuses or newborns [1]. The incidence is estimated to be about one in 2600 live births [2].

Fetal ovarian cysts are often described in the third trimester. The most plausible cause of this condition is represented by the excessive response of the fetal ovaries to high amounts of certain gonadotrophins and estrogens found in the placenta or maternal bloodstream [3]. Considering that very high β-hCG (Human Chorionic Gonadotropin) levels are known to be associated with alterations in maternal ovarian structure, extending causality to fetal ovarian cysts is only logical [4]. Diabetes, preeclampsia, and rhesus immunization are conditions frequently associated in pregnancies with ovarian fetal cyst [5]. In addition, as thyroid function can influence homeostasis leading to ovarian hyperstimulation in pregnant women, the elevated levels of TSH could mimic the effects of high β-hCG, explained by structural similarities between the above mentioned hormones but also of their receptors, prompting similar complications in the fetal ovary [6]. However, some authors suggest that a vascular anomaly is the underlying etiopathology behind an abnormal development of the primitive gonad [7].

Third trimester ultrasound usually detects most of the fetal ovarian cysts. There are two types of cysts: simple or complex. A simple cyst is most often considered a functional follicular cyst. It can be described by ultrasound using mode B as being anechoic, unilocular, round with a thin wall and a diameter superior to 2 cm [8]. By contrast, cysts with thick wall, heterogeneous content, anechoic content with hyperechoic components, intracystic septations and are considered to be complex cysts and can often lead to complications [8].

When a complex fetal ovarian cyst is suspected, a scrupulous evaluation of the urogenital tract and of the gastrointestinal tract should be performed to rule out frequently associated anomalies.

Differential diagnosis considered are: mesenteric cyst, fetal enteric duplication cyst, urachal cyst, omental cyst, meconium pseudocyst, dilated fetal renal pelvis or part of the ureter, dilated fetal loop of bowel, and hydrocolpos.

A pathognomic feature for the ovarian origin of a fetal cyst is “daughter cyst” sign that designates the presence of a secondary smaller cyst in the cavity of the primary cyst [5].

While the use of 3D imaging and color Doppler in fetal cerebral and cardiac evaluation is already well established [9], a rather recent application in fetal ultrasound diagnosis brings promising new input for assessment of surgical approach in ovarian cysts larger than 5 cm [8].

When fetal position, oligohydramnios or maternal habitus make ultrasound inadequate, magnetic resonance imaging examination (MRI) is an accurate alternative for prenatal evaluation of fetal structures [10]. Even though the current management of fetal ovarian cysts does not routinely include MRI, its use could bring forward a better algorithm for diagnosis.

Despite the progress from recent years, most of the cases of fetal ovarian cysts available in the literature are either case reports or short series of cases therefore there is no guideline available, making treatment reliant on the particular medical center inside approach [11].

Most of the patients are asymptomatic at birth; the treatment is usually based on the size and appearance of the cyst on ultrasound. Considering that they pose a high risk of complications such as intracystic hemorrhage, cyst rupture, birth dystocia and adjacent structures pressure (in large cysts) and even neoplastic transformation in complex cysts, surgical approach is applied in selected cases based on the individual risk evaluation [7,12].

In the present paper we provide an overview of our experience of fetuses with the suspicion of an antenatal ovarian cyst. We render the imaging diagnosis, management, and postnatal outcome of these patients.

## 2. Materials and Methods

The study included 19 cases of fetal ovarian cysts diagnosed prenatally in the Maternal and Fetal Medicine Department of 1st Clinic of Obstetrics and Gynecology Cluj-Napoca, and 9 patients referred to 1st Clinic of Obstetrics and Gynecology, Craiova, Emergency County Hospital, Romania. The cases were selected between 2013 and 2020. The inclusion criteria were female fetuses detected prenatally with unilateral or bilateral cystic masses, located in the lower fetal abdomen, with smooth, isolated, or heterogeneous internal structure, with no peristaltic, suspected to be of ovarian origin.

In order to ensure the completeness of prenatal and postnatal information, patients referred for second opinion that did not deliver in the above mentioned clinics were excluded from the study.

The study protocol was approved by the local ethics committees.

The data regarding the gestational age at the point of diagnosis, prenatal management and postnatal outcome were collected retrospectively from the patient’s electronic charts or by telephone from the mothers. Data on the postnatal evolution of the children were obtained directly from the mothers. Informed consent was obtained from all subjects, according to the World Medical Association Declaration of Helsinki, revised in 2000, Edinburgh.

We prenatally took into consideration the imaging aspect by ultrasound and MRI of the fetal pelvic masses, accompanying malformation, and mother’s medical and obstetric history for a possible etiological explication and pregnancy evolution. Postnatally, we focused on the imaging confirmation of the prenatal diagnosis, neonate’s immediate outcome, the indications leading to surgery, surgical methods employed and pathological aspect of the surgically removed materials.

**Figure 1:**
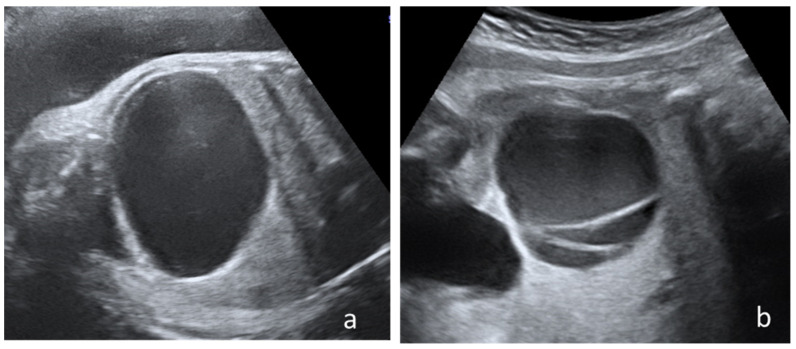
Cyst’s classification according to Nussbaum criteria: (**a**) Simple cyst with thin wall and anechoic aspect, (**b**) complex cyst with intracystic septation.

All patients were examined at the hospital admission by experienced physicians certified in Maternal and Fetal Medicine, using high-resolution ultrasound devices equipment Voluson E10 or Voluson 730 Expert (GE Healthcare Austria GmbH & Co OG.) available during that period, following a standardized protocol The Nussbaum criteria [13] were used to define the cysts type. Therefore, a fetal ovarian cyst is defined as simple if it is completely anechoic and has a thin wall (Figure 1a). By contrast the cyst is defined as complex if it is multilocular or unilocular with echogenic content, clot retraction clot, fluid-debris level (Figure 1b).

The delivery method was determined by obstetrical indication.

Neonates diagnosed prenatally with a complex cyst were examined in the first week after birth by a pediatric surgeon and radiologist. The neonates were treated surgically or referred to followed-up by transabdominal ultrasonic examination regular.

### Statistical Analysis

The data were compiled into a database using Microsoft Excel^®^ 2021 (version 16.56 purchased online) and the results were exposed using descriptive statistic. Continuous data were expressed as means and percentages.

## 3. Results

A total of 28 patients were registered in both hospitals with the diagnosis of fetal ovarian cysts. Four out of 28 patients were excluded from our study as they were referred to the Maternal and Fetal Medicine Department only for a second opinion or MRI investigation. Another three patients were lost to postnatal follow-up and, therefore, excluded from the study. Finally, 21 patients fulfilled all the inclusion criteria.

The demographic and clinical characteristics of the patients are depicted in Table 1.

The mean gestational age at the time of initial diagnosis was 31.28 weeks of gestation (WG) [range = 23 to 38 WG (SD ± 0.62)]. Most of the cysts were considered simple—13 cases and eight had a complex appearance. All cysts were unilateral and mostly detected in the right ovary (11/21 patients).

### 3.1. Cases with Spontaneous Prenatal Resolution (N = 5)

Five out of 21 cysts regressed completely during pregnancy without postnatal complications.

The median gestational age at diagnosis of the five cysts resorbed prenatally was 30.4 WG (range 23 to 36 WG). One patient that presented prenatal resolution of the fetal cysts was diagnosed with severe preeclampsia.

All cysts with prenatal resolution were isolated, mainly left ovarian cysts, three simple cysts with smooth walls and two complex cysts. The mean size of the largest diameter was 3.62 cm (SD ± 0.87). The longest period of prenatal detection of a fetal ovarian cyst during prenatal surveillance was 16 weeks (from 23 WG to 39 WG) in one primigravida, with otherwise physiologic pregnancy. Generally, the mean period of prenatal detection of the fetal ovarian cysts during prenatal surveillance was 8.3 weeks (SD ± 4.67).

### 3.2. Postnatal Evolution of the Neonates Who Underwent Surgery (N = 11)

Eleven out of the 21 infant’s required surgical treatment that was carried out in the first two weeks after birth. The mean size of the cysts that required surgical treatment was 6.48 cm (SD ± 1.89).

**Figure 2:**
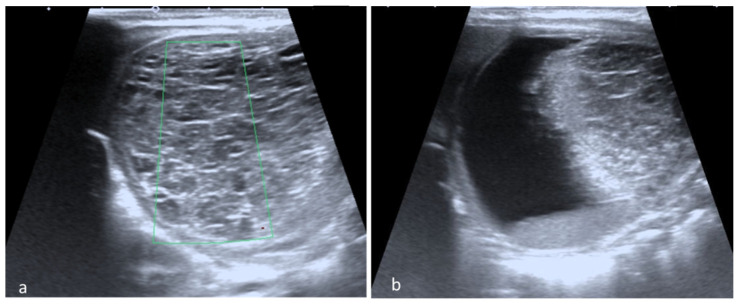
Ovarian torsion. (**a**) Postnatal abdominal ultrasound with enlarged, inhomogeneous structure without vascular Doppler signal; and (**b**) a cyst with fluid-fluid level.

The surgical approach was necessary after complications were suspected following ultrasound or fetal MRI, especially for complex cysts. The main surgical indication was ovarian torsion that was confirmed in eight cases, all with right ovarian cysts. We acknowledge that seven children with confirmed ovarian torsion did not have an acute abdomen at birth (Figure 2). Only one infant presented surgical abdomen four days after birth. In another three children, surgery was performed following the worrying increase in size of the ovarian cyst and intracystic bleeding during the first seven days after birth.

Interesting ovarian torsion was encountered in cases that presented relatively small diameter cysts (4 to 6 cm).

The surgical procedures performed were: laparoscopic cystectomy in four out of the 11 cases, classic salpingo-oophorectomy in five out of the 11 cases, one classic ovariectomy, and one classic cystectomy.

We mention one case diagnosed by prenatal ultrasonography with right ovarian fetal cyst complicated with intracystic bleeding and suspicion of torsion at 30 WG from a mother with gestational diabetes that associated left ovary agenesis diagnosed during the surgical procedures.

Histopathological exam of the annexes surgically removed revealed follicular cysts in majority of cases, one granulose cyst and one hemorrhagic cyst in one case with ovarian torsion. No malignant features were noted in the analyzed specimens.

### 3.3. Postpartum Evolution in the Children with Clinical Postnatal Follow-Up Recommendations (N = 5)

Considering the simple cysts ultrasound appearance and the lack of acute surgical abdomen at birth, five out of the 21 neonates with cysts with a mean size of 5.08 cm (±SD 1.2) were referred to postnatal follow-up clinically and by ultrasound.

**Figure 3:**
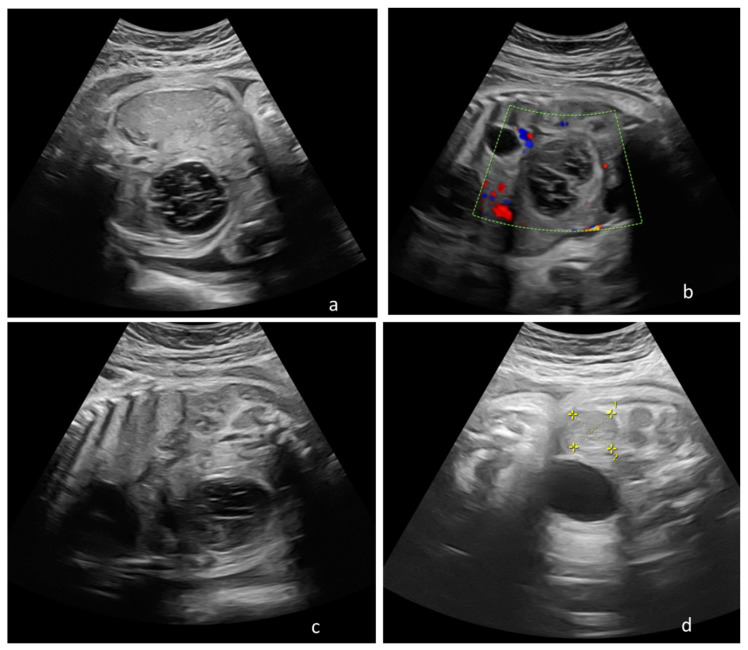
Consecutive prenatal ultrasonography depicting transformations of a fetal ovarian cyst that presented postnatal resorption, discovered at 31 weeks of gestation, and follow up until 36 weeks of gestation in a woman diagnosed with severe preeclampsia. (**a**) Thirty-one weeks of gestation female fetus with an 3 cm diameter echogenic right ovarian cyst with a thing wall, suggesting intracystic hemorrhage; (**b**) 32 weeks and 4 days, the changes of the cyst are followed, which becomes elongated, keeping the appearance of a intracystic hemorrhage, measuring 4.5/4 cm; (**c**) same case at 33 weeks and four days the fetal cystic begins to clarify its contents and maintains its decreases it diameter at 4.5/3.5 cm (**d**) same case at 36 weeks and four days, the fetal cyst is in important resorption measuring 2.32/2.39 cm. Figure 3a,c,d represent B-mode ultrasound images, while Figure 3b is CFM (Colour Flow Mapping) ultrasound mode that shows peripheral blood flow.

Still, three out of the five cases with recommended postnatal clinic and imagistic follow-up required emergency surgical treatment for ovarian torsion, intracystic hemorrhage, and peritonitis within the first six weeks after birth. In addition, in one case an exploratory surgical intervention for the suspicion of enteric duplication was performed, finding a self-amputated ovarian cyst, presence of which was suspected from week 31 of gestation. Only one case diagnosed antenatal with a simple 4 cm cyst presented spontaneous resorption within six months after delivery (Figure 3).

No cysts were treated by prenatal or postnatal aspiration.

### 3.4. The Role of Prenatal MRI for the Assessment of Fetal Ovarian Cysts in the Study Group

In cases that presented postnatal resolution, the diagnosis of fetal ovarian cyst and further resorption was confirmed by postnatal ultrasound examination of the ovaries or/and prenatal MRI.

The primary imagistic investigation used for the prenatal diagnostic and follow up of the cases was prenatal ultrasonography, but in five cases the investigations were completed with a prenatal MRI. The main indication for a prenatal MRI was to exclude an ovarian torsion or possible associated anomalies.

**Figure 4:**
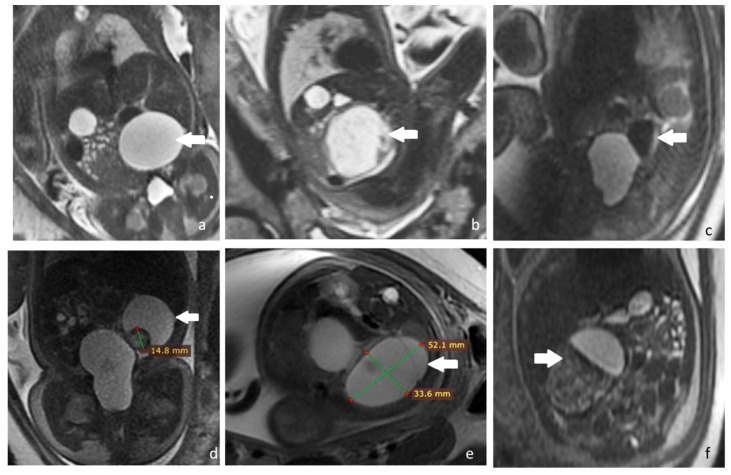
Fetal ovarian cyst-MRI aspects. All images are T2 SSFSE (single shot fast spin echo): (**a**) Simple ovarian cyst. Coronal view with a fluid filled cyst (arrow); (**b**) hemorrhagic cyst. Sagittal view with an inhomogeneous cyst, with hypointense fluid-fluid level (arrow); (**c**) hemorrhagic cyst. Sagittal view showing a cyst with hemorrhagic hypointense content and fluid-fluid level (arow); (**d**) daughter cyst. Coronal view showing a hemorrhagic daughter cyst in a larger fluid filled cyst (arrow); (**e**) septated ovarian cyst. Axial view showing a cyst with fine internal septations and uncomplicated fluid hyperintensity (arrow); (**f**) ovarian torsion. Enlarged ovary with inhomogeneous structure and a fluid-filled cyst, mimicking a fluid-fluid level (arrow).

Some of the prenatal IRM aspects (simple cyst, complex cysts, hemorrhagic cyst, and ovarian torsion) are depicted in Figure 4.

MRI correctly identified ovarian torsion in all cases and had an important contribution to the diagnosis of intracystic hemorrhagic changes and helped in establishing differential diagnoses.

## 4. Discussion

The study depicts our centers experience regarding the prenatal surveillance by ultrasound and/or MRI and postnatal outcomes of 21 patient’s diagnosed prenatally with ovarian cysts.

The majority of fetal ovarian cysts are benign, but the serious complications that arise, such as torsion, pose a life-threatening risk that usually is approached surgically [14].

Literature mostly places diagnosis gestational age in the last trimester [10,12] with very few cases being diagnosed in the second trimester as early 19 WG [15]. Our earliest detection was at 23 WG, this particular case having a complete resolution by 39 weeks.

Evaluation of fetal ovarian cysts in pregnancy should include thorough assessment of complications signs, such as hydramnios in partial obstruction of the gastrointestinal tract, ascites, tachycardia, intracystic hemorrhage, or torsion [14].

The differential diagnostics is an important step and factored out by identifying the organ of origin, observing the anatomy and placement of the cyst.

The most frequent differential diagnosis considered that can be described using prenatal ultrasonography are renal cysts (unilocular or multiple) that are found in the renal area, with or without alterations of the kidneys architecture. Other diagnosis that can be considered are hydronephrosis and ureterocele, often associated; the first being described as a mass adjacent to the vertebral column distorting the renal pelvis, the latter located by entry of the bladder. In addition, hydrocolpos, described as a mass posterior to the bladder often associated with fetal uterine dilatation, must be ruled out [2].

Gastrointestinal masses that can be taken into account (e.g., enteric duplication cyst, meconium pseudocyst, choledochal cyst) are often easily diagnosed as they attach to the respective organs and lack the signs of an ovarian cyst.

A surgical approach is often followed in spite of the high risk of neonatal surgery, in the hope of preservation of the ovary despite very few obtaining that result [5]. In addition, surgery is recommended in complex ovarian cysts, which pose a high risk of torsion and other complications, manifesting in various ways intrapartum depending on its size and nearby organs impact [14]. Often pediatric surgeons use laparoscopy in the neonate to help minimize the invasive procedure, especially if the surgery is recommended at birth [16].

In our lot of neonates, 11 required surgical treatment peripartum after undergoing prenatal imaging evaluations.

Approximately 40% of cases described in literature required emergency surgery for ovarian torsion, most of them manifesting clinical symptoms [14]. Still, in our study group only one neonate developed clinical signs of acute abdomen by day 4th of life. The majority of patients confirmed during the surgical exploration with ovarian torsion were without any clinical signs of surgical abdomen. The decision for surgery was based on in utero imagistic appearance of the ovarian mass and postnatal imagistic evaluation.

While torsion is usually a complication described in large complex cysts [12], in our lot the torsioned ovary presented relatively small cysts ranging between 4 to 6 cm diameters.

Conservative management is recommended in cysts under 20 mm diameter with no complications [16]. Almost 50% of ovarian cysts involute spontaneously pre- or postnatally [14]; therefore, a conservative management with ultrasound and clinical evaluations is usually considered.

In our observation, approximately a quarter of cases regressed completely during pregnancy. Interestingly, most of the prenatal cystic resolution were located in the left ovary and were of both complex and simple character.

To avoid surgical complications, antepartum percutaneous aspiration of ovarian cyst was tested. However, they stall to bring significant results to justify the risk of premature labor, intracystic bleeding or infection, while also presenting the possibility of recurrence due to the causal factor persisting. Despite this, it could be the solution to alleviate the symptoms and treat selected cases. Recommendations so far indicate that this procedure could be the preferred method in large cysts over 4 cm diameter that distended the fetal abdomen, are rapidly grown in their formation, or auto-amputated cysts free in the abdominal fetal cavity [6]. However, the procedure is not used in our medical center and as such none of the cases were subjected to percutaneous aspiration.

Some cases were selected for follow-up approach considering the ultrasound appearance and clinical state, but except for one case involving a simple 4 cm cysts (suspected as follicular) which resorbed by sixth month postpartum, all concluded with surgical emergency intervention.

Out of our selected sample of cases, all of the pregnancies were spontaneous, with the majority of them (61.9%) presenting no maternal pathologies before or during pregnancy.

Even though studies encourage natural birth in the absence of other aggravating factors [12], the general current approach is mainly based on personal medical experience. Over 80% of women in our study were selected to undergo cesarean section. Elective caesarian was required in two of our patients for preterm delivery complications.

Nonetheless, iatrogenic preterm delivery dependent on early signs of complications, such as increasing size or intracystic echoes, followed by prompt neonate surgery, could cause alternate issues for the child, while ovarian function might already be compromised [8].

One case that posed such significant impact on term-born child development was a complicated ovarian cyst, involving intracystic hemorrhage and torsion suspicion by the thirtieth week of gestation, which during peripartum surgery was found to be associated with left ovary agenesis.

In the absence of strict guidelines, medical decision regarding delivery method for fetuses with ovarian cysts is highly dependent on maternal option and the anticipated need for newborn surgery.

Histopathological examination is needed in order to assess the type of cysts and refer the patients to further investigations depending on the outcome. The majority of ovarian cysts found in fetuses and newborns are follicular or theca lutein in nature being related to hormonal imbalances. While teratoma or chistadenoma have been found, the incidence is low compared to hormone-related cysts [8]. Usually, complicated cysts, especially torsion related pathology, are hemorrhagic and pose a more significant risk of complications if spontaneous perforation occurs [12]. Malignant ovarian cysts are scarcely found in fetal and newborns, but, nevertheless, surgical intervention is advised if imaging raises a suspicion of malignancy in complex cysts [17].

Most of the cases in our study, at histopathological examination, presented with follicular cysts with only one granulose cyst and one hemorrhagic cyst, all complicated with ovarian torsion. This finding is in accord with known literature [12].

While ultrasound is regarded in most cases sufficient for assessing the evolution of fetal ovarian cysts, certain characteristic, such as torsion suspicions or age of intracystic hemorrhage, require fetal MRI [12]. The bulk of our cases were successfully managed by ultrasound evaluation during pregnancy and postpartum, with five of them also undergoing fetal MRI, which confirmed ovarian torsion and help initiate early peripartum surgery.

Although cystic size is considered to be strongly linked to complications [6] the cases presented in this study were of mean size 5.08 cm in follow-up group and in peripartum surgery group largest being of 6.48 cm. They ranged from simple in follow-up group to mixed (simple and complex) in peripartum surgery group without significant difference in evolution, as out of the entire lot of 21 cases, merely six resolved (only one postpartum) without surgical intervention, despite the different initial approach.

This raises the question whether, taking into consideration the limitation because of lack of percutaneous aspiration in our group, surgical approach could realistically be avoided in cases with persistence of cysts postpartum. It also casts doubt of size truly being a factor for complications, as some studies also found relatively small cysts (mean 45 mm) presenting with hemorrhagic infarction [14].

An obvious limitation of this study besides the small number of cases included is the retrospective character. The small number of cases can be explained by the strict case selection criteria, including only cases that have given birth in our centers.

However, we are open to the possibility of future studies with the inclusion of several obstetrics centers in order to be able to solve a uniform management protocol of these cases based on the experience and possibilities of each center.

## 5. Conclusions

Given the hormone-dependence of the majority of these cysts, spontaneous resolution is to be expected; however, as our study found, presence of the ovarian mass in relation to the rapid development of the fetus and infant could lead to complications, if resolution does not occur in due time.

## Figures and Tables

**Table 1:** Demographic and clinical characteristics of the patients included in the study.

Characteristic	Number of Cases
**Mean maternal age**	29.95 (SD ± 4.41) years
**Associated maternal pathologies and treatment during pregnancy**	
- No maternal pathology	13 (61.9%),
- Gestational diabetes	2 (9.5%), diet, no insulin
- Hypothyroidism	2 (9.5%); Levothyroxine 50–75 UI
- Preeclampsia	2 (9.5%), Methyldopa 250–750 mg/day
- Other non-metabolic pathology	2 (9.5%)
**Insufficient prenatal care**	
- (Less than 3 prenatal consultations)	4 (19.04%)
**Obstetrical history**	
- Spontaneous pregnancy	100%
- Prenatal genetic screening using combined test	15 (71.42%) normal, 6 (28.5%) ND
- Morphologic scan at 19–23 weeks	19 (90.47%) no major structural defects detected
- More than 2 hospitalizations during pregnancy	3 (14.28%)
**Delivery**	
- Spontaneous vaginal birth	4 (19.04%)
- Cesarean section (CS)	17 (80.95%), 7 elective and 10 emergencies
- Term delivery	18 (85.7%)
- Preterm delivery	3 (14.28%), 2 CS by medical indication
ND = not done; CS = caesarian section	

## Data Availability

The data that support the findings of this study are available from the corresponding author upon reasonable request.

## References

[1] Crombleholme T.M., Craigo S.D., Garmel S., D’Alton M.E. (1997). Fetal ovarian cyst decompression to prevent torsion. J. Pediatr. Surg..

[2] Trinh T.W., Kennedy A.M. (2015). Fetal Ovarian Cysts: Review of Imaging Spectrum, Differential Diagnosis, Management, and Outcome. RadioGraphics.

[3] Tyraskis A., Bakalis S., Scala C., Syngelaki A., Giuliani S., Davenport M., David A., Nicolaides K., Eaton S., De Coppi P. (2018). A retrospective multicenter study of the natural history of fetal ovarian cysts. J. Pediatr. Surg..

[4] Berezowski A.T., Machado J.C., Mendes M.C., Moura M.D., Duarte G., Cunha S.P. (2001). Prenatal diagnosis of fetal ovarian hyperstimulation. Ultrasound Obstet. Gynecol..

[5] Dimitraki M., Koutlaki N., Nikas I., Mandratzi T., Gourovanidis V., Kontomanolis E., Zervoudis S., Galazios G., Liberis V. (2011). Fetal ovarian cysts. Our clinical experience over 16 cases and review of the literature. J. Matern.-Fetal Neonatal Med..

[6] Hershman J. (2004). Physiological and pathological aspects of the effect of human chorionic gonadotropin on the thyroid. Best Pract. Res. Clin. Endocrinol. Metab..

[7] Enríquez G., Durán C., Torán N., Piqueras J., Gratacós E., Aso C., Lloret J., Castellote A., Lucaya J. (2005). Conservative Versus Surgical Treatment for Complex Neonatal Ovarian Cysts: Out-comes Study. Am. J. Roentgenol..

[8] Bascietto F., Liberati M., Marrone L., Khalil A., Pagani G., Gustapane S., Leombroni M., Buca D., Flacco M.E., Rizzo G. (2017). Outcome of fetal ovarian cysts diagnosed on prenatal ultrasound examination: Systematic review and meta-analysis. Ultrasound Obstet. Gynecol..

[9] Zaharie G.C., Hasmasanu M., Blaga L., Matyas M., Muresan D., Bolboaca S.D. (2019). Cardiac left heart morphology and function in newborns with intrauterine growth restriction: Relevance for long-term assessment. Med. Ultrason..

[10] Muresan D., Popa R., Stamatian F., Rotar I. (2015). The use of modern ultrasound tridimensional techniques for the evaluation of fetal cerebral midline structures—A practical approach. Med. Ultrason..

[11] Nemec U., Nemec S.F., Bettelheim D., Brugger P.C., Horcher E., Schöpf V., Graham J.M., Rimoin D.L., Weber M., Prayer D. (2012). Ovarian cysts on prenatal MRI. Eur. J. Radiol..

[12] Akın M.A., Akın L., Ozbek S., Tireli G., Kavuncuoglu S., Sander S., Akcakus M., Gunes T., Ozturk M.A., Kurtoglu S. (2010). Fetal-Neonatal Ovarian Cysts-Their Monitoring and Management: Retrospective Evaluation of 20 Cases and Review of the Literature. J. Clin. Res. Pediatr. Endocrinol..

[13] Nussbaum A., Sanders R., Benator R., Haller J., Dudgeon D. (1987). Spontaneous resolution of neonatal ovarian cysts. Am. J. Roentgenol..

[14] Heling K.-S., Chaoui R., Kirchmair F., Stadie S., Bollmann R. (2002). Fetal ovarian cysts: Prenatal diagnosis, management and postnatal outcome. Ultrasound Obstet. Gynecol..

[15] Meizner I., Levy A., Katz M., Maresh A.J., Glezerman M. (1991). Fetal ovarian cysts: Prenatal ultrasonographic detection and postnatal evaluation and treatment. Am. J. Obstet. Gynecol..

[16] van Niekerk M.L. (2008). Ovarian cysts in infants: Indications for intervention and advantages of the minimally invasive method. SAJ Child Health.

[17] Matthews M.A., Raval M., Watkins D.J., King D. (2014). Diagnosis and management of an ovarian cyst complicated by torsion in utero: A case report. J. Pediatr. Surg. Case Rep..

